# Pilot Randomised Trial of Calibrated Formula Volumes to Optimise Growth in Infants at Risk for Overweight: The Califormula Randomised Clinical Trial

**DOI:** 10.1111/ijpo.70141

**Published:** 2026-08-03

**Authors:** Ian M. Paul, Bernardo L. Horta, Mina Desai, Michael G. Ross

**Affiliations:** ^1^ Penn State College of Medicine Hershey Pennsylvania USA; ^2^ School of Medicine Universidade Federal de Pelotas Pelotas Brazil; ^3^ The Lundquist Institute at Harbor‐UCLA Medical Center Torrance California USA

**Keywords:** growth, infant formula, overweight

## Abstract

**Background:**

Formula feeding and having a mother with overweight are associated with overweight during infancy.

**Objectives:**

We sought to pilot test calibrated formula feeding guidance using age and weight specific intake recommendations to improve weight outcomes amongst at‐risk infants.

**Methods:**

Exclusively formula fed newborns with birthweight ≥ 50th percentile delivered to mothers with pre‐pregnancy body mass index (BMI) ≥ 25 kg/m^2^ were randomised to intervention and usual care groups. At monthly visits from 1 to 5 months, mothers of intervention group infants with weight‐for‐length (WFL) ≥ 75th percentile were advised to give 10% less daily formula than required based upon sex, age and weight. The primary outcome was conditional weight gain (CWG) between birth and 6 months. Secondary outcomes included WFL *z*‐score and overweight (WFL ≥ 95th percentile) at 6 months, as well as growth trajectory.

**Results:**

Sixty infants were enrolled; 36 mothers (66.7%) had pre‐pregnancy BMI ≥ 30. Seventeen (28.3%) withdrew before 6 months. At each assessment, a minority had WFL ≥ 75th percentile required to receive calibrated formula guidance. There were no group differences in CWG (intervention mean 0.27, 95% CI −0.20 to 0.75 vs. control mean −0.12, 95% CI −0.62 to 0.37; *p* = 0.25) or for secondary outcomes.

**Conclusions:**

Calibrated infant formula recommendations were not associated with growth outcomes.

**Trial Registration:** Clinicaltrials.gov identifier: NCT05104073 (registered October 11, 2021)

## 1 Introduction

Infancy is a critical period of developmental plasticity with lasting metabolic and behavioural consequences [1]. Obesity is one of the outcomes influenced by this plasticity during infancy. The obesity epidemic is a public health crisis, nearly one in five US children aged 2–19 are living with obesity, and approximately one in three are living with overweight (body mass index [BMIs] ≥ 95th and 85th percentiles, respectively) [2, 3]. For many children, this problem begins early as 23% of 2–5 years olds are already overweight [3] and 9%–14% are already obese [2, 4]. Primary prevention is likely the best solution, as a recent simulation study suggested that ‘a 2‐year‐old who is obese is more likely to be obese at 35 years of age than an overweight 19‐year‐old’ [5].

There is evidence that formula feeding is associated with a modestly increased risk of obesity [6, 7, 8, 9, 10] and rapid infant weight gain [11], with further evidence that the volume of formula consumed, overfeeding, is an important contributor to excess weight gain [12, 13] as might be expected. Similar findings have been reported for bottle feeding breast milk and formula [14]. Adjusting or calibrating the recommended amount of formula across the first 6 months after birth shows promise as a strategy to reduce rapid infant weight gain and early life overweight [13]. Interestingly, recent data supporting such a strategy were reported describing that while larger infants consume higher daily volumes of formula than smaller infants (mL/day), they consume relatively less in proportion to their weight (mL/kg/day) [15].

We conducted the Califormula pilot randomised trial to determine if formula feeding recommendations that are calibrated using age and weight specific caloric intake recommendations can prevent excessive infant weight gain and reduce overweight in the first 6 months after birth amongst infants born to mothers with overweight prior to pregnancy electing to exclusively formula feed their infants. Calibrated formula feeding refers to adjusting the recommended daily caloric formula intake to account for weight status. We hypothesised that the calibrated formula intake intervention will reduce rapid infant gain and overweight during infancy resulting in lower weight‐for‐length (WFL) at age 6 months. In this pilot, we additionally sought to determine the feasibility of giving tailored formula recommendations to parents as well as the acceptability of this guidance as measured by parental adherence to formula volume recommendations.

## 2 Materials and Methods

### 2.1 Participants and Study Design

**FIGURE 1:**
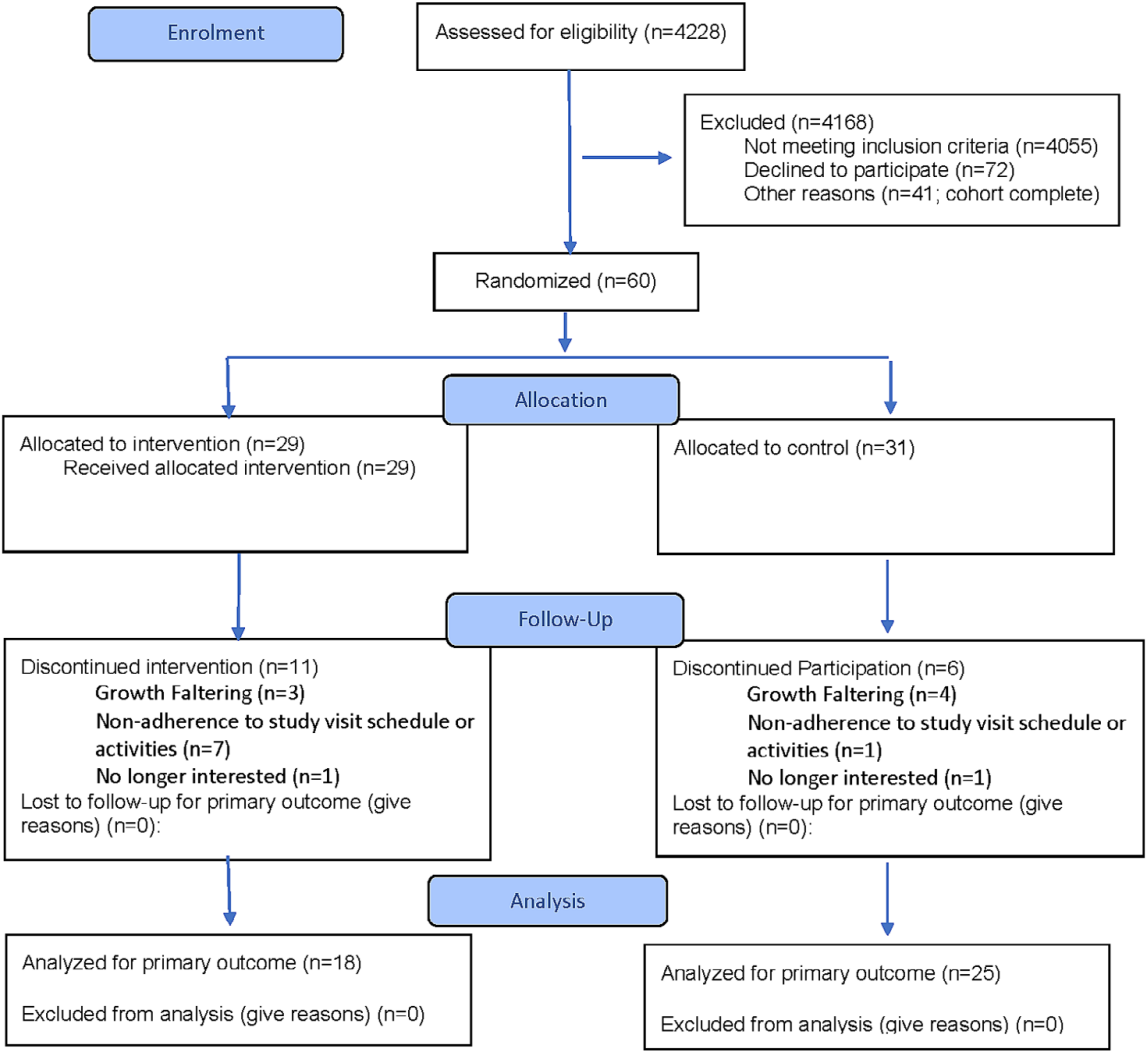
CONSORT 2025 flow diagram.

This pilot study was approved by the Penn State Institutional Review Board (STUDY00018788). All participating mothers provided written informed consent. Mothers and their newborns were recruited after delivery from a single maternity ward at Penn State Health Children's Hospital (Hershey, PA) or at affiliated Penn State Health clinics between birth and age 1 month (Figure 1). The enrolment period spanned from 25 January 2022 to 9 October 2023. Infants were eligible for inclusion in the trial if they met the following criteria: gestational age ≥ 37 weeks, birthweight ≥ 50th percentile according to the 2013 Fenton growth charts [16], maternal pre‐pregnancy BMI ≥ 25 kg/m^2^, age ≤ 1 month, maternal age ≥ 18 years, English‐speaking mother, absence of any substantial neonatal morbidity that could affect feeding or weight gain and an intention to exclusively formula feed.

The sample size for this pilot study was based upon the number of participants that we felt we could feasibly recruit given the time and resources available for recruitment. As this pilot sought to determine feasibility, 60 participants seemed adequate for this purpose with the potential to determine the sample size necessary for a fully powered subsequent trial.

Participant groups were randomly allocated to intervention or control using a computer‐generated randomisation programme generated by an independent statistician. Randomisation was stratified for sex of the infant (male/female) and maternal pre‐pregnancy BMI category (25–29.9 vs. ≥ 30 kg/m^2^) to ensure balanced distribution across these key variables. Infants in the intervention group were expected to visit the clinic monthly until age 6 months to be weighed and measured. Parents were instructed to complete diary cards recording volume of infant formula consumed for the 3 days prior to the visits. The diary card gathered information on volume consumed in each feeding for the day, type of infant formula, size of bottle used and other sources of nutrition (e.g., juice, water, foods). Control infants followed the same procedures, but did not have scheduled visits at ages 3 and 5 months. Instead, their visits corresponded to usual care well child visits at ages 1, 2, 4 and 6 months. Age 6‐month assessments were completed by March 2024.

### 2.2 Intervention

At the age 1‐month visit, intervention group parents were provided with educational handouts on hunger and satiety cues, a digital streaming copy of the video, *Happiest Baby on the Block* [17], and two videos on bottle feeding including responsive bottle‐feeding practices [18, 19]. For those with WFL < 75th percentile, research staff provided parents with a recommendation for total daily formula volume to be consumed based upon energy requirement (kcal/kg/day) research conducted by Butte et al. that accounts for sex, weight and current age [20]. To provide guidance for what parents should expect over the next month, the child's WFL percentile was reviewed. Parents were informed of the volume to expect at the subsequent monthly visit with the expectation that the child would maintain the same weight‐for‐age percentile. For those with WFL ≥ 75th percentile, recommended current daily formula volumes were calculated based upon the child's current age, weight and energy requirement (kcal/kg/day) with a 10% reduction in recommended daily volume. Parents were also informed of the recommended volume to expect at the subsequent visit in 1 month, again with a 10% reduction from what the current weight‐for‐age percentile would be if maintained for another month. Ten percent was chosen as the amount to reduce for this pilot because it balances feasibility (relatively likely that parents could comply) and safety (unlikely to cause failure‐to‐thrive by reducing total daily formula volume by ~3 oz). Lastly, parents were given instruction on formula preparation. Specifically, they were asked to prepare the formula volume in each bottle on the lower end of what they anticipated the infant will eat. For example, if the infant typically consumed 3–4 oz per feed, they were instructed to prepare 3 oz first. If hunger cues persist, they would then prepare an additional 1–2 oz of formula. Similar procedures were followed for the intervention group at subsequent visits. However, if the child's WFL percentile was ≥ 75th percentile for a second consecutive month, the recommended adjustment in daily formula volumes was decreased by 15%.

### 2.3 Measures

Information on demographic variables was collected at enrolment, whereas maternal age, pre‐pregnancy weight and height, delivery mode, gestational age, birth weight and birth length were gathered from electronic medical records. Self‐identified race and ethnicity were collected using categories consistent with National Institutes of Health enrolment tables as required by the funding agency. Additional survey data were collected regarding pregnancy complications (e.g., diabetes mellitus), cigarette smoking, education, marital status, insurance type, government assistance (e.g., food stamps) and infant formula use.

For anthropometric measurements, weights were measured at outpatient clinic visits for both study groups to the nearest 0.001 kg using a calibrated, digital electronic scale. Recumbent lengths were measured to the nearest 0.1 cm using a portable stadiometer (Shorr). Weight, length and weight/length *z*‐scores, according to age and sex, were estimated using the World Health Organization (WHO) growth standards [21]. Anthropometric measurements were used to calculate the growth‐related adverse events related to growth faltering using two criteria: (a) WFL < 5th percentile for sex and age on the WHO growth chart and (b) downward crossing of > 2 major centile lines on the WHO growth chart after enrolment.

### 2.4 Outcomes and Statistical Analysis

The primary outcome for this pilot study is CWG between birth and age 6 months using methods described by Griffiths et al. as a measure of rapid infant weight gain [22]. CWG is calculated by regressing weight for age *z*‐score at 6 months on birthweight and earlier measures of weight and length, and length at 6 months; standard residual was estimated. The conditional variable indicates weight gain in the first 6 months. We explored the moderating role of sex, delivery mode and maternal parity on intervention effects. As a secondary outcome, similar analyses were conducted for the intervention period spanning ages 1–6 months. Additional secondary outcomes include cross‐sectional comparisons of mean WFL *z*‐score at age 6 months and the proportion of infants with overweight (WFL ≥ 95th percentile on WHO growth standards [21]) at 6 months. We used Stata 19 for the analyses. Statistical significance was defined as a *p*‐value < 0.05. Means were compared using analysis of variance, and the chi‐squared test was used to compare proportions. To assess growth trajectories of the study groups, we fit a repeated measures analysis of variance of WFL. We further sought to examine the acceptability of providing formula volume recommendations to parents by assessing parent adherence to formula volume recommendations as measured by 3‐day feeding diaries at 2, 3, 4, 5 and 6 months for the intervention group. Analyses followed intention‐to‐treat principles, analysing participants by randomised study group.

## 3 Results

A total of 60 infants were enrolled into the study at a mean (SD) age of 14.8 (8.3) days. This included 34 females (56.7%) and 26 males (43.3%), with 21 (35.0%) delivered via Caesarean section and 39 (65.0%) delivered vaginally (Table 1). Mean (SD) gestational age was 38.8 (0.9) weeks, and mean (SD) birthweight was 3683 (321) g. Twenty‐nine were randomised to the intervention group and 31 to control, with no significant baseline or demographic differences between groups. Mean (SD) maternal age at delivery was 29.2 (5.2) years, and 36 (66.7%) had pre‐pregnancy BMI of 30 or greater. The majority (79.3%) were multiparous and self‐described as White (75%) and non‐Hispanic (85%). Although the prevalence of obesity was similar between groups, mothers in the intervention group had a higher mean (SD) pre‐pregnancy BMI than controls [35.1 (7.2) vs. 31.7 (4.2); *p* = 0.03]. No other maternal baseline or demographic variables were significantly different between groups. Consistent with their intent at enrolment, no infants received any breast milk during the study period.

**TABLE 1:** Demographic and baseline characteristics by study group.

	Intervention, *N* = 29	Control, *N* = 31
Infant		
Sex, *N* (%)		
Male	14 (48.3)	12 (38.7)
Female	15 (51.7)	19 (61.3)
Gestational age, mean (SD), weeks	38.7 (0.9)	38.8 (1.0)
Birthweight, mean (SD), g	3711 (295)	3657 (346)
Birthweight percentile, mean (SD)	80.2 (12.8)	76.3 (11.4)
Birth length, mean (SD), cm	52.0 (2.32)	51.5 (2.00)
Delivery mode, *N* (%)		
Vaginal	17 (58.6)	22 (71.0)
Caesarean	12 (41.4)	9 (29.0)
Mother		
Age at delivery, mean (SD), years	28.1 (4.6)	30.1 (4.6)
Pre‐pregnancy BMI category, *N* (%)^a^		
25.0–29.9 kg/m^2^	9 (31.0)	11 (35.5)
≥ 30 kg/m^2^	20 (69.0)	20 (64.5)
Gestational weight gain, mean (SD), kg	13.8 (7.0)	12.7 (8.6)
Parity, *N* (%)		
1	9 (31.0)	4 (12.9)
2	11 (37.9)	8 (25.8)
3	4 (13.8)	11 (35.5)
≥ 4	5 (17.2)	8 (25.8)
Cigarette use during pregnancy, *N* (%)	4 (13.8)	4 (12.9)
Diabetes during pregnancy, *N* (%)	6 (20.7)	7 (22.6)
Race, *N* (%)		
Black	3 (10.3)	6 (19.4)
White	21 (72.4)	24 (77.4)
Other	5 (17.3)	1 (3.2)
Hispanic/Latino ethnicity, *N* (%)	5 (17.2)	4 (12.9)
Maternal education, *N* (%)		
Some high school	1 (3.5)	4 (12.9)
High school graduate	18 (62.0)	12 (38.6)
Some college	6 (20.7)	7 (22.6)
Completed college	2 (6.9)	6 (19.4)
Postgraduate college	2 (6.9)	2 (6.5)
Living with a partner, *N* (%)	23 (79.3)	23 (74.2)
Insurance type, *N* (%)		
Private	12 (41.4)	13 (41.9)
Medicaid	16 (55.1)	18 (58.1)
None	1 (3.5)	0 (0.0)

^a^ Mean (SD) pre‐pregnancy BMI: intervention group vs. control—35.1 (7.2) versus 31.7 (4.2); *p* = 0.03.

Amongst the cohort of 60 infants enrolled, 17 (28.3%) withdrew prior to completing the 6‐month study follow‐up including 7 (3 intervention, 4 control) that experienced the adverse event of growth faltering during the intervention period. The attrition rate was similar according to birthweight percentile, sex, maternal age and maternal pre‐pregnancy BMI. In the intervention group, 37.9% of the children did not complete the age 6‐month follow‐up, whereas 19.4% in the control group did not complete that assessment (*p* = 0.11).

Per the study protocol, guidance for calibrating formula volume was given only to intervention group participants when WFL was ≥ 75th percentile at monthly assessments from ages 1 to 5 months. At each time point, a minority of intervention group participants received calibrated formula guidance (Table 2). For the first month exceeding this threshold, a 10% reduction in daily formula volume was recommended. There were 12 intervention group infants with consecutive months having WFL ≥ 75th percentile prompting the recommendation for a 15% reduction in formula volume.

**TABLE 2:** Participants with weight‐for‐length ≥ 75th percentile at each assessment.^a^

Assessment age (months)	Intervention group, *N* (%)	Control group, *N* (%)	*p*
1	12/29 (41)	12/30 (40)	0.91
2	6/24 (25)	15/29 (52)	0.048
3	11/20 (55)	N/A	
4	8/20 (40)	9/26 (35)	0.71
5	10/18 (56)	N/A	

^a^ Control infants did not have scheduled visits at ages 3 and 5 months.

### 3.1 Primary Outcome of CWG From Birth Through 6 Months

**FIGURE 2:**
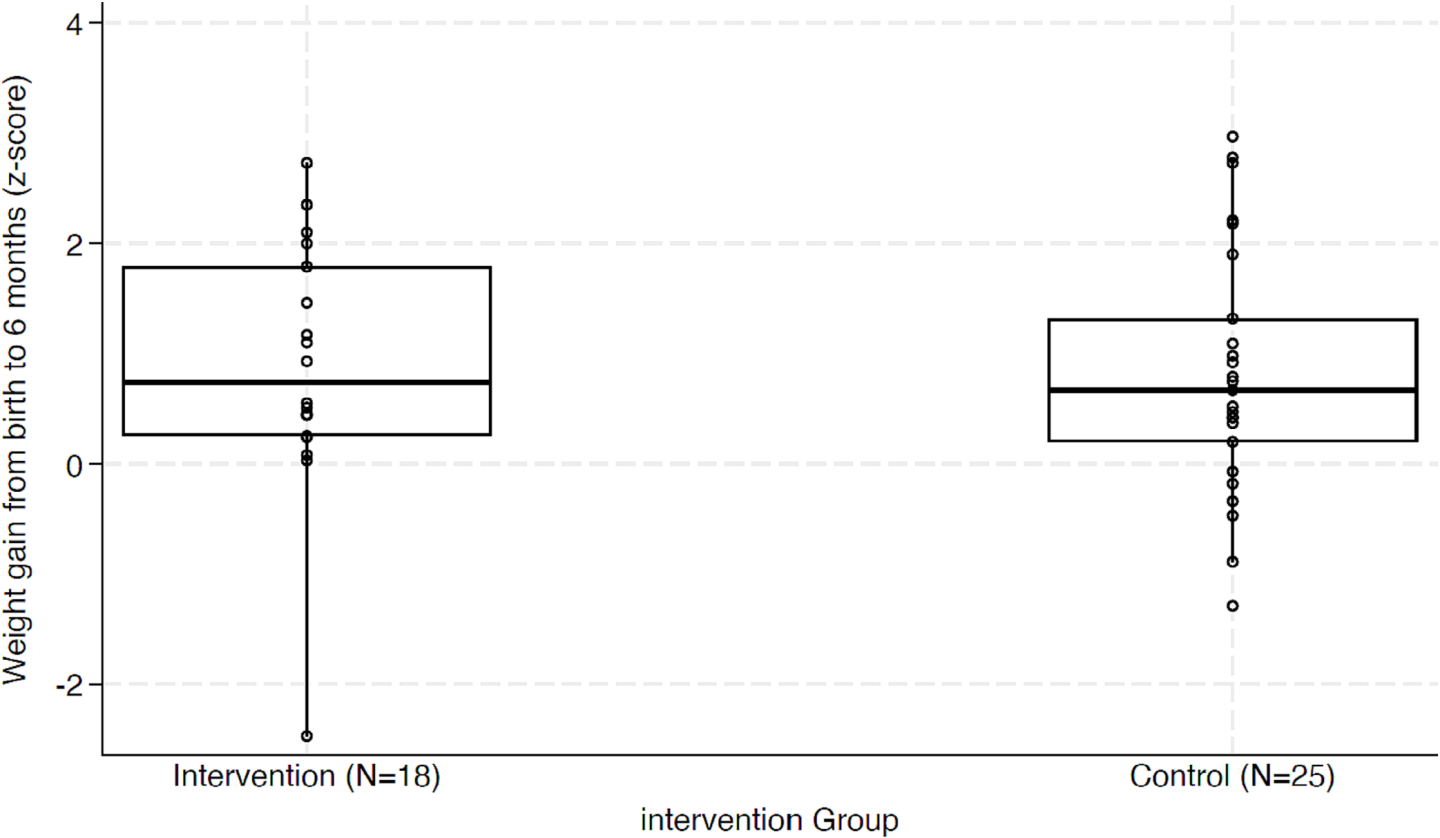
Weight gain in *z*‐scores between birth and 6 months by study group.

During the first 6 months, there was no significant difference in weight gain as assessed by CWG between study groups (intervention mean 0.27, 95% CI −0.20 to 0.75 vs. control mean −0.12, 95% CI −0.62 to 0.37; *p* = 0.25). Figure 2 shows the distribution of CWG scores for both study groups.

### 3.2 Secondary Growth Outcomes

During the intervention period of 1–6 months, CWG again did not differ between study groups (intervention mean 0.28, 95% CI −0.20 to 0.77 vs. control mean −0.14, 95% CI −0.63 to 0.35; *p* = 0.22). We also compared the mean WFL *z*‐scores between groups at 6 months, again with no difference between intervention versus control (0.73 ± 0.57 vs. 0.65 ± 0.38; *p* = 0.81). At age 6 months, 4 of 22 (22.2%) of intervention group participants were overweight compared with 2 of 25 (8%) controls (*p* = 0.18). A repeated measures analysis to evaluate growth trajectories between 1 and 6 months revealed no difference in mean (95% CI) WFL between groups (12.4 [12.1–12.7] vs. 12.1 [11.8–12.3]; *p* = 0.92).

### 3.3 Acceptability of Intervention

Across the 6 months of the study, daily formula volume consumed by infants was similar between groups at each assessment point (Table 3). The proportion of infants with WFL ≥ 75th percentile ranged from 20.7% to 61.1% in the intervention group and 34.4% to 64.0% in the control group. Given that this was a pilot study, we sought to determine whether intervention group parents with infants ≥ 75th percentile would adhere to the reduced formula volume recommendations. Unfortunately, analyses restricted to those children generally revealed no differences between groups, suggesting that caregivers were unable to reduce the amount of formula that they gave to their infant.

**TABLE 3:** Feeding frequency and formula volume by study group at study assessment timepoints.

Visit age, *N*	Daily feeds, mean (SD)			Daily formula volume, oz, mean (SD)		
	Intervention	Control	*p*	Intervention	Control	*p*
Before 1 month, 52	7.1 (1.7)	6.6 (1.1)	0.16	26.6 (7.6)	25.4 (5.1)	0.52
After 1 month, 53	7.0 (1.7)	6.5 (1.3)	0.20	28.3 (8.2)	25.3 (5.6)	0.12
Before 2 months, 51	6.2 (1.4)	5.3 (1.0)	0.02	29.1 (7.6)	26.5 (5.1)	0.15
After 2 months, 45	6.2 (1.4)	5.6 (1.4)	0.12	29.9 (7.5)	26.7 (5.8)	0.11
Before 4 months, 44	5.6 (1.3)	5.0 (1.1)	0.11	31.6 (7.3)	29.5 (6.0)	0.30
After 4 months, 43	5.4 (1.3)	5.0 (1.2)	0.30	30.5 (6.2)	30.6 (8.8)	0.97
Before 6 months, 43	5.8 (1.4)	4.4 (1.2)	0.001	30.4 (6.8)	29.7 (7.4)	0.77
Analyses restricted to those children with weight‐for‐length ≥ 75th percentile in the previous visit						
Before 1 month, 17	7.8 (1.3)	6.6 (1.4)	0.13	25.7 (3.7)	26.2 (5.4)	0.85
After 1 month, 18	7.4 (0.7)	6.3 (1.0)	0.03	27.0 (3.4)	25.0 (5.2)	0.40
Before 2 months, 21	6.6 (1.7)	5.7 (1.3)	0.16	29.0 (4.1)	28.2 (5.4)	0.69
After 2 months, 20	6.5 (1.6)	6.0 (1.8)	0.53	28.7 (3.5)	29.1 (5.7)	0.84
Before 4 months, 19	5.1 (0.8)	5.1 (1.3)	0.98	33.5 (14.0)	31.2 (7.1)	0.64
After 4 months, 19	5.0 (1.0)	5.3 (1.3)	0.71	31.6 (11.6)	33.0 (10.4)	0.81
Before 6 months, 16	6.3 (1.4)	4.6 (1.2)	0.02	31.5 (2.0)	28.5 (3.7)	0.07

## 4 Discussion

As demonstrated in multiple animal studies [23, 24], offspring of mothers with overweight and obesity demonstrate early life hyperphagia and rapid weight gain, a consequence in part of increased hypothalamic appetite neurons and reduced satiety neurons. It is therefore not surprising that maternal pre‐pregnancy weight is strongly predictive of childhood obesity. As such, for this study we enrolled a higher‐risk cohort of infants with birthweights over the 50th percentile delivered to mothers with pre‐pregnancy overweight or obesity who planned to exclusively formula feed. We attempted to reduce early life caloric intake in our intervention by limiting formula volumes. Limiting newborn nutrition (e.g., cross‐fostering to artificial large litter sizes) prevents excessive early life weight gain and may reduce rates of adult obesity [25, 26, 27]. While reducing formula volume and secondarily caloric intake seemed like a logical approach to prevent excessive infant weight gain during the first 6 months after birth, the results of this study demonstrated that applying this strategy in the real world is challenging. Even amongst a higher risk cohort, only a minority of intervention participants met our pre‐defined threshold of WFL ≥ 75th percentile at each timepoint, and caregivers appeared to have difficulty in limiting formula intake compared with controls, even when guidance to limit formula volume was coupled with responsive feeding information. All study outcomes related to formula intake, including daily feeds and daily volumes, were similar between study groups making the lack of intervention effects on weight outcomes unsurprising.

Reducing milk or formula volume as a method to slow weight gain clearly works in the animal laboratory setting [28]. However, in the real world setting for humans, even sophisticated, multi‐layer behavioural interventions to slow infant weight gain generally are unsuccessful [29]. While instructing caregivers to reduce formula volume with detailed guidance seemed logical, other approaches to limit infant caloric intake appear necessary to slow high‐risk infant weight gain. The study's attrition rate posed additional challenges with 12% meeting our definition of ‘growth faltering’, perhaps an unintended consequence of our infant selection criteria. At birth, participating infants had birthweights averaging above the 75th percentile. Given natural regression to the mean tendencies, these infants are more likely to experience downward crossing of > 2 major centile lines on the growth chart than those born near the bottom of the chart.

As for alternative approaches that could be attempted, recently Abrahamse‐Berkeveld and colleagues tested a novel infant formula where the size of the protein‐coated lipid droplets was increased to better simulate human milk [30]. Compared with a standard infant formula that contained small protein‐coated lipid droplets as is typical for commercially available formulas, infants randomised to the novel formula had lower BMI at the age 1 year primary outcome timepoint, a finding that continued through the age 5‐year follow‐up. Importantly, those fed the novel formula grew similarly to a breastfed reference group. The mechanism by which this modified formula impacted infant weight gain is unknown, though it may include infant taste and appetite, nutrient absorption and metabolism and microbiome alterations, amongst others.

Infant formulas will always be compared with human milk, a complex biological fluid of more than 200 identified components [31] including solutions, colloids, membranes, globules and live cells [31]. Specifically, human milk contains, on average, 0.8%–0.9% protein, 3%–5% fat, 6.9%–7.2% carbohydrates and 66 kcal/100 mL [32, 33, 34, 35] with an interquartile range 62.0–72.5 kcal/100 mL, reflecting the significant individual variance of caloric content. Milk fat content, which contributes 40%–50% of calories, increases by fourfold and caloric content by twofold from foremilk to hindmilk during a single feed, while additional variation occurs with time of day, weeks postpartum and in response to maternal dietary modifications. It would be expected that both the single feed and diurnal fat/caloric content variation impact infant appetite and satiety and thus modulate breast milk intake, ultimately impacting growth. Despite the natural variance in breastmilk, infant formulas are relatively similar between brands and by commercial necessity, have the same composition at every feed for every infant consuming them. It is possible that the lack of formula composition‐induced satiety signals limited caregiver adherence to volume reduction in our study.

This single centre pilot trial was limited by our ability to ensure caregiver adherence to the formula recommendations. Indeed, the results showing no difference in daily formula volumes between groups indicate that we could not fully test the hypothesis that reduced formula volumes would result in slower weight gain amongst this high‐risk cohort. Further, while rates of adverse events were similar between groups, because adherence to the reduced formula volume recommendations was poor, we cannot guarantee that the safety of interventions designed to reduce caloric intake may prevent excessive infant weight gain.

## 5 Conclusion

While calibrating formula volume to limit rapid weight gain theoretically could prevent overweight during infancy, implementation of a protocol to accomplish this proved challenging in the real world. Our pilot trial found no differences between study groups related to infant growth during the first 6 months suggesting that the intervention in its current form is ineffective. Future studies might implement additional strategies to increase adherence and/or manipulate the components of the formula rather than the volume in order to prevent rapid infant weight gain and overweight amongst high‐risk infants.

## Funding

This work was supported by grants R21HD104028 from the Eunice Kennedy Shriver National Institute of Child Health and Human Development and UL1TR002014 from the National Center for Advancing Translational Sciences.

## Conflicts of Interest

Ian M. Paul discloses honoraria from Danone North America related to this work. The other authors declare no conflicts of interest.

## Supporting information


**Data S1:** CONSORT 2025 checklist item description.

## Data Availability

The data that support the findings of this study are available from the corresponding author upon reasonable request.
